# Effective strategies for preventing and managing workplace’s burnout

**DOI:** 10.1192/j.eurpsy.2026.10265

**Published:** 2026-07-31

**Authors:** S. Medved

**Affiliations:** University Clinical Hospital Zagreb, Zagreb, Croatia

## Abstract

**Abstract:**

Stress and interpersonal relationships in the clinical setting greatly impact the well-being of early-career psychiatrists (ECP). Key sources of stress are often related to patient care and suffering, organizational and system-level inefficiencies, workload, unpredictable schedules, and financial strain. Research highlights systemic stressors such as staffing shortages, administrative burden and obstacles, and a lack of resources, as well as individual stressors related to competence, career stagnation, work-life balance, and personal difficulties. Therefore, strategies for reducing burnout include both individual and structural approaches. We will provide an overview of the most common sources of stress, their impact on ECPs’ well-being and propose viable solutions.

**Disclosure of Interest:**

None Declared

